# Effect of morphologies and compositions of silver-based multicomponent heterogeneous nanocrystals on the reduction of 4-nitrophenol†

**DOI:** 10.1039/d3na00473b

**Published:** 2023-08-30

**Authors:** Ming-Shiuan Huang, Hsien-Tai Cheng, Su-Wen Hsu

**Affiliations:** a Department of Chemical Engineering, National Cheng Kung University, Taiwan No. 1 University Road, East Dist. Tainan City 70101 Taiwan ROC swhsu@gs.ncku.edu.tw

## Abstract

Silver-based nanocrystals have excellent catalytic performance in various reactions, such as the reduction of 4-nitrophenol. The catalytic performance of nanocrystals varies with several parameters, including nanocrystal morphology, composition, and plasmon-induced hot electrons around nanocrystals. Here, highly heterogeneous nanocrystals (Au–Ag and Ag_2_S–Ag nanocrystals) fabricated on polymer films *via* a seed-mediated method are used as catalysts for the reduction of 4-nitrophenol, and the effect of the morphology and composition of nanocrystals on the catalytic performance is investigated. These nanocrystals on polymer films exhibit higher reusability (low catalyst loss) in catalytic applications compared to catalysts dispersed freely in the reaction solution. The excellent catalyst performance of these heterogeneous nanocrystals is attributed to their high surface area/volume ratio (flower-like nanocrystals) and strong synergistic effect (cage-like nanocrystals). These nanocrystals with special morphologies and composites showed higher catalytic performance (higher reactivity at lower catalyst contents) than silver-based nanocrystals reported in the literature. Due to the excellent plasmonic properties of Ag nanocrystals, the catalytic performance of these nanocrystals can be further enhanced by generating hot electrons around the nanocrystals under irradiation. These results demonstrated that by carefully controlling the morphology and composition of nanocrystals, it is possible to design and fabricate excellent catalysts for various reactions.

## Abstract 2

Ag-based bi-component nanocrystals (Ag–Au and Ag–Ag_2_S nanocrystals) fixed on a polymer matrix as catalysts for 4-NP reduction exhibited high reactivity can reusability compared with Ag nanocrystals.

## Graphical abstract

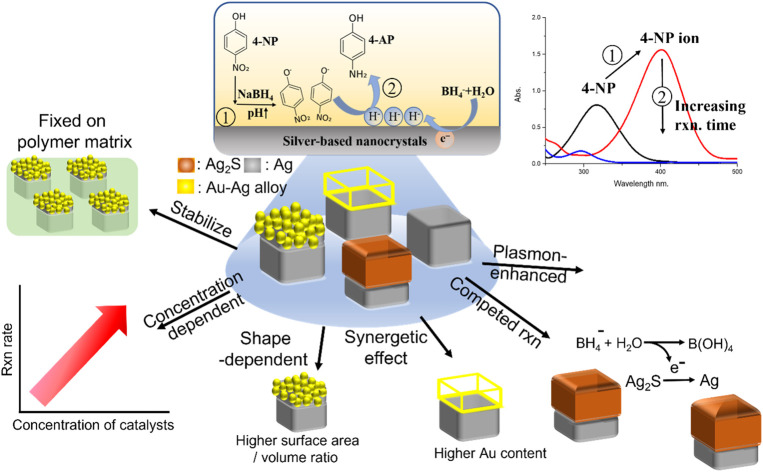

## 1. Introduction

Due to the increasingly serious environmental pollution, converting toxic substances in water, such as toxic aqueous phase organic wastes, into low-toxicity recyclable products has become a hot research topic.^1–4^ One of the most common difficult-to-treat water pollutants and environmentally harmful materials was 4-nitrophenol (4-NP), which had been demonstrated to be highly toxic and carcinogenic. Interestingly, the reduction product of 4-NP, 4 aminophenol (4-AP), is a valuable material due to its low toxicity and various useful applications, such as corrosion inhibition, anticorrosion lubrication, drying agent, photographic development, *etc.*^5–7^ To covert trace amounts of 4-NP in water to valuable 4-AP, cost-effective, stable, and highly active catalysts for 4-NP reduction were highly desirable. The most commonly used materials as catalysts for the reduction of 4-NP to 4-AP were Ag, Au, Pt, and Ag_2_S nanocrystals.^8–17^ The advantages of Ag nanocrystals as catalysts were: (1) catalytic activity can be tuned by controlling their size and shape; (2) the reaction temperature requirement was low; (3) the practical application price was relatively low; (4) relatively low toxicity for environmental applications.^13,18–20^ Even though Ag nanocrystals were ideal catalysts for 4-NP reduction, Ag nanocrystals used as catalysts still had several issues such as easy aggregation and lower catalytic activity, which needed to be treated to improve their catalytic performance. The aggregation problem can be effectively reduced by incorporating Ag nanocrystals into substrates, such as polymer matrices.^21^ The lower catalytic activity can be improved by using (1) anisotropic Ag nanocrystals, such as Ag nanocubes (AgNCs), and (2) bimetallic Ag nanocrystals to replace the monometallic Ag nanocrystals.^9,15^

**Fig. 1:**
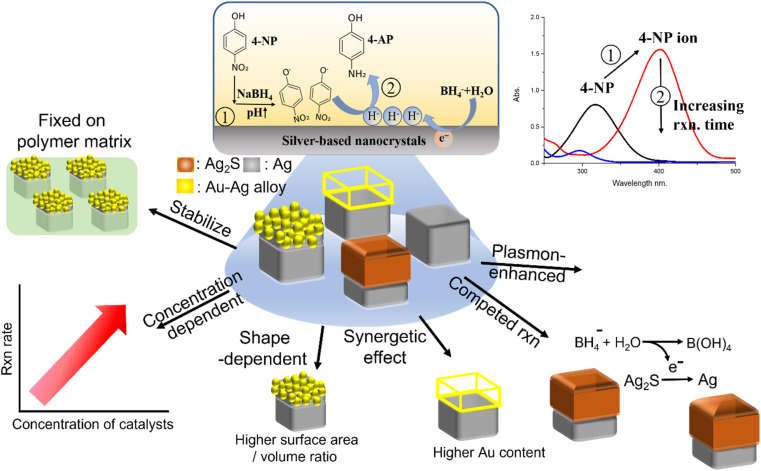
Schematic of silver-based heterogeneous nanocrystals as catalysts for the reduction of 4-nitrophenol (4-NP). There are various catalytic results that characterized the catalytic performance of catalysts: reusability, concentration-dependent, shape-dependent, synergetic effects, composition-dependent, and the plasmon enhancement effect (photocatalyst).

Recently, our group fabricated highly heterogenous bicomponent nanocrystals on AgNCs embedded in a polymer matrix as templates. Here, a hydrophobic layer, such as a polystyrene film, can act as an interface to suppress the formation of bi-component nanocrystals in aqueous solution, leading to the formation of highly heterogeneous nanocrystals with controllable morphologies and components of nanocrystals. These highly heterogeneous bicomponent nanocrystals synthesized on the hydrophobic layer also showed that these nanocrystals can be well dispersed on a polymer matrix and can also be used to address the problems of aggregation^21^ and low activity as catalysts for the 4-NP reduction reaction, resulting in excellent catalytic performance. The hydrophobic layer can also prevent the adsorption of 4-NP molecules in the matrix from affecting the evaluation of the reduction reaction and prevent the loss of heterogeneous nanocrystals during the recycling process after the reduction reaction of 4-NP molecules in aqueous solution. To systematically study the catalytic performance of these silver-based bicomponent nanocrystals as 4-NP reduction catalysts, several parameters were used to characterize the catalytic performance, as listed below: (1) reusability tests by comparing catalysts that were freely dispersed in the reaction solution or fixed on a polymer matrix; (2) the catalyst concentration-dependent reaction rate of 4-NP reduction; (3) the catalyst shape-dependent reaction rate of 4-NP reduction (involving the effects of the surface area/volume ratio, synergetic effect, and competitive reactions); (4) the catalyst component-dependent reaction rate of 4-NP reduction; (5) the plasmon-enhancement effect (as shown in Fig. 1).

The results showed that the reusability of silver-based nanocrystals fixed on a polymer matrix as catalysts was higher than that of catalysts freely dispersed in the reaction solution. Compared with the pristine AgNCs fixed in a polymer matrix, the flower-like Au–AgNC and cage-like Au–AgNC nanocrystals fixed in a polymer matrix had higher 4-NP reduction reactivity, which can be attributed to the high surface area/volume ratio and strong synergetic effect, respectively. However, compared with the pristine AgNC fixed in a polymer matrix, the core–shell Ag_2_S–AgNC nanocrystals fixed in a polymer matrix exhibited lower 4-NP reduction reactivity and reshaped nanocrystals, which may be caused by the reduction of Ag ions on Ag_2_S to Ag atoms, a competitive reaction of 4-NP reduction. Due to the excellent plasmonic properties of Au–AgNC nanocrystals, Au–AgNC nanocrystals can generate many “hot electrons” around them under irradiation in plasmonic mode. These hot electrons can be used to accelerate the reaction rate of the 4-NP reduction. Compared with the reduction of 4NP without irradiation assistance, the reduction rate of 4-NP exhibited an enhancement factor of about 1.1–1.25 times under irradiation assistance, and the enhancement factor increased with the number of hot electrons (the number of catalysts). These results provided a comprehensive understanding of the possible factors that lead to changes in the reduction reactivity of 4-NP when Ag-based heterogeneous nanocrystals were used as catalysts. This information paved the way for designing excellent catalysts for the 4-NP reduction reaction.

## 2. Results and discussion

To systematically study the catalytic performance of silver-based nanocrystals as 4-NP reduction catalysts, silver-based bicomponent nanocrystals with different morphologies, and compositions (flower-like Au–AgNCs, cage-like Au-AgNCs, and core–shell Ag_2_S–AgNC nanocrystals) were used as catalysts for NaBH_4_-assisted reduction of 4-NP in aqueous solution. Since the catalytic performance was affected by several parameters, the results were divided into several subsections with different variables to discuss the influence on the catalytic performance: (A) reusability of catalytic performance: catalysts were freely suspended or fixed on the polymer matrix; (B) the amount of catalyst used; (C) shape of bicomponent nanocrystals: flower-like Au–Ag nanocrystals (higher surface area/volume ratio) or cage-like Au–Ag nanocrystals (stronger synergistic effect); (D) component of bicomponent nanocrystals: Au–Ag nanocrystal or Ag_2_S–Ag nanocrystals; (E) irradiation assistance.

### 2.1 Reusability of catalytic performance: catalysts were freely suspended or fixed on the polymer matrix

**Fig. 2:**
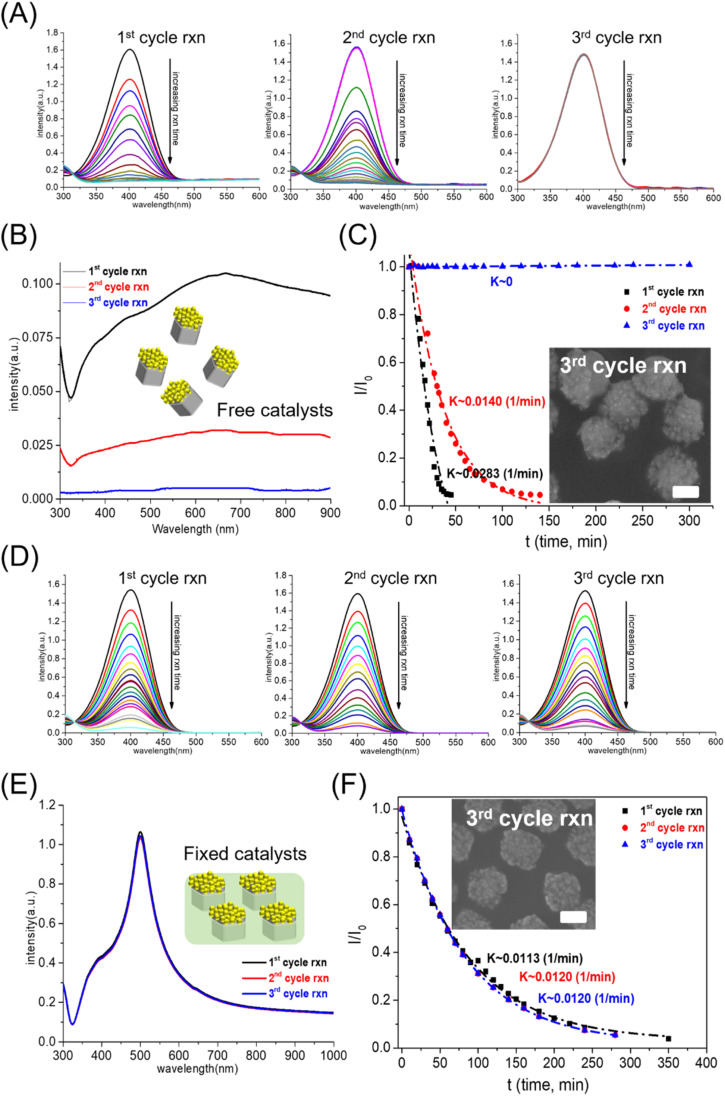
Reusability tests of flower-like Au–AgNC nanocrystals as catalysts for the reduction of 4-NP. (A) For the nanocrystals freely suspended in the reaction solution, the concentration of 4-NP varied with the reaction time during 1–3 cycles. (B) The extinction spectra of flower-like Au–AgNC nanocrystals in liquid during 1–3 cycles. (C) Changes in 4-NP (*I*/*I*_0_) concentration exhibited an exponential relationship with reaction time during 1–3 cycles of the 4NP reduction reaction. SEM image of Au–Ag nanocrystals after 3 cycles of the 4NP reduction reaction as an inset image. (D) For the nanocrystals fixed in a polymer matrix, the concentration of 4-NP varied with the reaction time during 1–3 cycles of the 4NP reduction reaction. (E) The extinction spectra of flower-like Au–AgNC nanocrystals fixed in a polymer matrix during 1–3 cycles of the 4NP reduction reaction. (F) Changes in 4-NP (*I*/*I*_0_) concentration exhibited an exponential relationship with reaction time during 1–3 cycles of the 4NP reduction reaction. SEM image of Au–Ag nanocrystals after 3 cycles of the 4NP reduction reaction as an inset image. The scale bar was 100 nm.

The first problem with solid catalysts for water pollutant treatment was reusability: the washing step in the reused process led to a decrease in the amount of solid catalyst, which reduced the catalytic performance. Our group synthesized silver-based bicomponent nanocrystals on a template substrate, consisting of an AgNC array partially embedded in a polymer matrix.^21,22^ The PS matrix served as a heterogeneous interface for controlling the morphology and composition of bicomponent nanocrystals synthesized by a seed-mediated method: AgNCs as seeds partially embedded in the PS matrix as templates to generate the bi-component nanocrystals. The as-made silver-based bicomponent nanocrystals fixed in a polystyrene matrix can be used to eliminate the loss of nanocrystals during the washing process. The reusability of Ag-based nanocrystals as catalysts for 4-NP reduction can be characterized by the catalytic performance of 4-NP reduction in multi-cycle reactions. The catalytic performance can be characterized by the variation of 4-NP concentration (absorption peak at 400 nm) with reaction time. For flower-like Au–AgNC nanocrystals freely suspended in the reaction solution, the rate of change of 4-NP concentration with the reaction time decreased significantly with the increase of the number of reaction cycles (as shown in Fig. 2(A)). This can be attributed to the decrease in the amount of flower-like Au–AgNC nanocrystals in the reaction solution during the washing process, as shown in the extinction spectra of flower-like Au–AgNC nanocrystals in one to three reaction cycles in Fig. 2(B). This result indicated that the reusability of freely dispersed nanocrystals as catalysts was poor.

However, the reusability of nanocrystals as catalysts can be efficiently enhanced by fixed flower-like Au–AgNCs in a polystyrene matrix, as shown by the changes in the 4-NP concentration in the multi-cycle test in Fig. 2(D). The extinction spectra of flower-like Au–AgNC nanocrystals in a polystyrene matrix showed that the number of nanocrystals remained unchanged during the multi-cycle test as shown in Fig. 2(E). The concentration of 4-NP (relative intensity of the absorption peak at 400 nm, *I*/*I*_0_) had an exponential decay relationship with the reaction time, indicating that the kinetic mechanism of using freely suspended nanocrystals and fixed nanocrystals as catalysts for the 4-NP reduction was the first-order reaction as shown in Fig. 2 (C), (F), and ESI Table S1.† And the reaction rate (reaction constant, *K*) of 4-NP reduction in both cases also confirmed that the reusability of fixed nanocrystals as catalysts was higher than that of freely dispersed nanocrystals as catalysts.

### 2.2 The amount of catalyst used

**Fig. 3:**
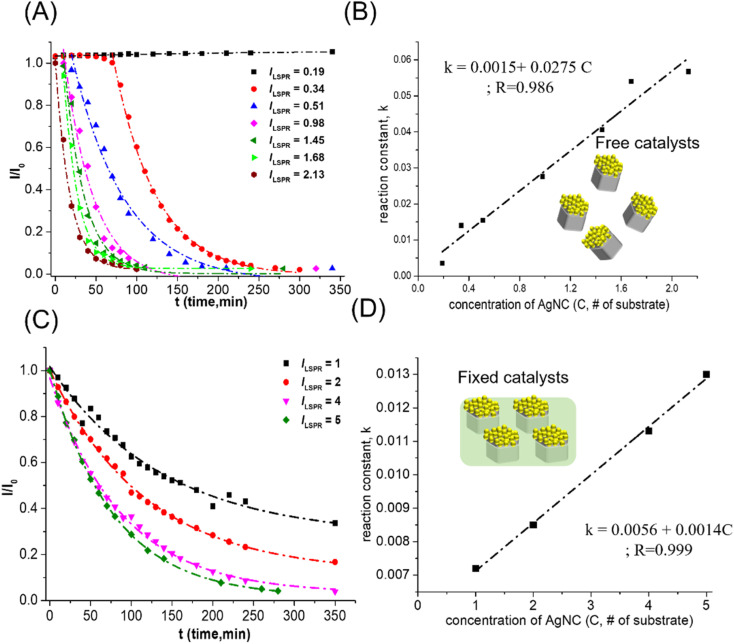
Changes in the reactivity of 4-NP reduction with different concentrations of flower-like Au–AgNC nanocrystals as catalysts. (A) For the nanocrystals freely suspended in the reaction solution, the concentration of 4-NP varied with reaction time at different nanocrystal concentrations. The dashed line was generated by fitting the curve with an exponential equation: 
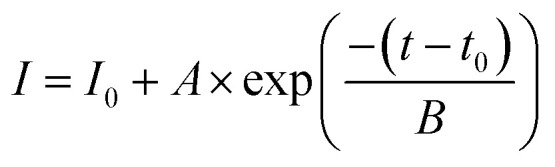
, here *I*: the concentration of 4-NP, *I*_0_: the concentration of 4-NP at the start of the reaction and 1/*B*: reaction constant (*k*). (B) The rate of the 4-NP reduction reaction (reaction constant) varied with the concentration of Au–AgNCs (*C*, corresponding to the # of substrates, Au–AgNC array in a polymer matrix, releasing in the solution). The relationship between *k* and *C* was linear. (C) For the nanocrystals fixed in a polymer matrix, the concentration of 4-NP varied with reaction time at different nanocrystal concentrations. The dashed line was generated by fitting the curve with an exponential equation. (D) the rate of the 4-NP reduction reaction (reaction constant) varied with the concentration of Au–AgNCs (C, # of substrates, Au–AgNC array in a polymer matrix). The relationship between *k* and *C* was linear.

The other important factor for changes in the reaction rate of 4-NP reduction was the concentration of catalysts in the reaction solution. To compare the concentrations of freely dispersed Au–Ag nanocrystals and fixed Au–Ag nanocrystals for 4-NP reduction catalysts, the concentration of Au–AgNCs was calculated from the number of 1 cm × l cm substrates. After the release of Au–AgNC nanocrystals from the polystyrene matrix, the plasmon resonance intensity in the extinction spectrum of freely dispersed Au–AgNC nanocrystals can be used to quantify the amount relative to the 1 cm × l cm substrate with fixed Au–AgNC nanocrystals. For different concentrations of flower-like Au–AgNC nanocrystals as 4-NP reduction catalysts, the change of 4-NP (*I*/*I*_0_) concentration with reaction time was accelerated with the increase of catalyst concentration in the reaction solution as shown in ESI S1† and Fig. 3(A). And the kinetic mechanism of 4-NP reduction maintained a first-order reaction with the change of catalyst concentration, *I*/*I*_0_ decaying exponentially with reaction time, as shown in ESI Table S2.† The concentration of freely dispersed flower-like nanocrystals as the 4-NP reduction catalysts exhibited a linear relationship with reduction rate, as shown in Fig. 3(B). Similar catalytic performance, linear reduction rate with catalyst concentration, and kinetic reaction mechanism, first-order reaction, can be observed in fixed flower-like Au–AgNC nanocrystals as 4-NP reduction catalysts, as shown in Fig. 3(C), (D), and ESI S2 and Table S3.† However, freely dispersed nanocrystals as catalysts showed higher reduction reactivity than fixed nanocrystals as catalysts. This result was because the overall reaction surface area (# of activation sites) of freely dispersed nanocrystals for 4-NP reduction was higher than that of fixed nanocrystals for 4-NP reduction. For freely dispersed flower-like nanocrystals as the 4-NP reduction catalysts, the 4-NP reduction did not start immediately, and the delay time increased with the decrease of freely dispersed flower-like nanocrystal concentration (as shown in Fig. 3(A)). The delay in the reduction of 4-NP can be attributed to the adsorption of water molecules on the catalyst surface, resulting in the need to replace the water molecules on the catalyst surface with 4-NP and NaBH_4_ for 4-NP reduction.^23^ And the decreases in the catalyst concentration led to an increase in the time required for diffusion of 4-NP and NaBH_4_ to reach the catalyst surface, resulting in a further delay of the 4-NP reduction.^24^ However, for fixed Au–Ag nanocrystals for 4-NP reduction catalysts, the 4-NP reduction occurred immediately after NaBH_4_ injection, which resulted from the fixed Au–Ag nanocrystals being stored in the air, resulting in no adsorption of water molecules on the catalyst surface for 4-NP reduction.

### 2.3 Shape of bicomponent nanocrystals: flower-like Au–Ag nanocrystal (higher surface area/ volume ratio) or cage-like Au–Ag nanocrystal (stronger synergistic effect)

**Fig. 4:**
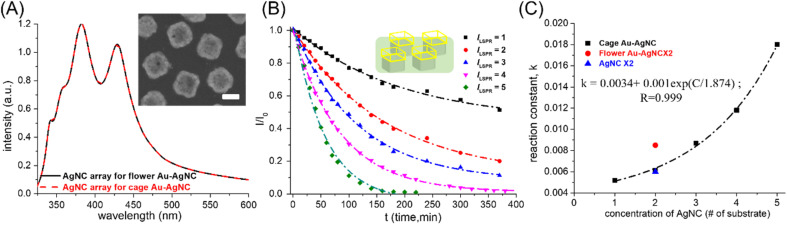
Changes in the reactivity of 4-NP reduction with different concentrations of cage-like Au–AgNC nanocrystals as catalysts. (A) Extinction spectra of the AgNC array in a polystyrene matrix, which were used to fabricate flower-like Au–AgNC (black curve) and cage-like Au–AgNC (red curve). SEM image of cage-like Au–Ag nanocrystals as an inset image. (B) The concentration of 4-NP varied with reaction time at different nanocrystal concentrations. The dashed line was generated by fitting the curve with an exponential equation. (C) the rate of the 4-NP reduction reaction (reaction constant) varied with the concentration of Au–AgNCs (*C*, # of substrates, Au–AgNC array in a polymer matrix). *k* exhibited an exponential growth relationship with the increase of *C*. The reaction rate of 4-NP reduction was compared between pristine AgNCs (blue triangle) and flower-like Au–AgNC nanocrystals (red circle). The scale bar was 100 nm.

Another parameter that affected the catalytic performance of Au–AgNC nanocrystals as 4-NP reduction catalysts was the morphology of nanocrystals. Here, the flower-like Au–AgNC nanocrystals and cage-like Au–AgNC nanocrystals were used as 4-NP reduction catalysts to study the shape-dependent catalytic performance of nanocrystals. The concentration of the two different morphologies of Au–AgNC nanocrystals can be characterized by measuring the intensity of the extinction spectrum of the AgNC array on a polystyrene matrix, which was used to synthesize Au–AgNC nanocrystals. The concentration of the AgNC array in the 1 cm × l cm substrate used for the synthesis of flower-like Au–AgNC nanocrystals (black curve of the extinction spectrum in Fig. 4(A)) was similar to that for the synthesis of cage-like Au–AgNC nanocrystals (red curve of the extinction spectrum in Fig. 4(B)), the concentration of flower-like Au–AgNC nanocrystals in the substrate can be considered to be the same as that of cage-like Au–AgNC nanocrystals. The reduction rate of 4-NP with two flower-like Au–AgNC substrates as catalysts (red circle dot in Fig. 4(C) and ESI S4†) was higher than that of two cage-like Au–AgNC substrates as catalysts (black square dot in Fig. 4(C) and ESI S4†). This result was caused by the fact that flower-like Au–AgNC nanocrystals had more activation sites (huge surface area/volume ratio) than cage-like Au–AgNC nanocrystals. Even though the surface area of cage-like Au–AgNC nanocrystals was smaller than that of pristine AgNCs (as theoretical calculation in supporting information S6†), the reduction rate of 4-NP with two cage-like Au–AgNC substrates as catalysts was similar to that of two pristine AgNC substrates (blue triangle dot in Fig. 4(C) and ESI S4†). This can be attributed to the abundant Au–Ag alloy in the cage-like nanocrystal, which led to the enhanced “synergetic effect “created between Au and Ag atoms. This strong “synergetic effect “can result in an increase in the reactivity of 4-NP reduction, as shown in ESI S5(B).†

The kinetic mechanism of using fixed cage-like Au–AgNC nanocrystals as catalysts for the 4-NP reduction reaction also exhibited first-order reaction (the 4-NP concentration decayed exponentially with reaction time) over a wide range of nanocrystal concentrations as shown in Fig. 4(B) and ESI S3 and Table S4.† However, the reduction rate (reaction constant, *K*) of 4-NP increased exponentially with the number of substrates with cage-like Au–AgNC nanocrystals (as shown in Fig. 4(C)), which was clearly different from the linear relationship between the reduction rate of 4-NP and the number of substrates with flower-like Au–AgNC nanocrystals. This deviation should be attributed to the compositional difference between flower-like Au–AgNCs (Au atom deposition on AgNCs) and cage-like Au–AgNCs (the Au–Ag alloy frame on the top of Ag nanocrystals),^21^ which led to different reactivity of 4-NP reduction. For flower-like Au–AgNCs (Au atom deposition on AgNCs), the 4-NP reduction mainly occurred on the petals (Au atoms) of flower-like Au–AgNCs, which led to a linear increase in the reaction rate (reaction constant) with the increase in the number of catalysts. However, cage-like Au–AgNCs (the Au–Ag alloy frame on the top of Ag nanocrystals) and two different reaction sites (Au–Ag alloy frame and Ag nanocrystals) can be used to reduce 4-NP and led to different reactivities. At a low concentration of cage-like Au–AgNCs, 4-NP reduction can occur at both reaction sites. As the concentration of cage-like Au–AgNCs increases, the 4-NP reduction preferentially occurred on the Au–Ag alloy frame, which led to a significant increase in 4-NP reduction reactivity (exponential increasing) due to the “synergetic effect” between Au and Ag atoms. This deviation should be attributed to the stronger “synergetic effect” between Au and Ag atoms for 4-NP reduction in cage-like Au–AgNC nanocrystals than in flower-like Au–AgNC nanocrystals (the atomic content of Au in cage-like Au–AgNC nanocrystals was 8.8 times higher than in flower-like Au–Ag nanocrystals, as shown in ESI S5†). And with the increase in cage-like Au–AgNC concentration (# of the substrate), the “synergetic effect” of 4-NP reduction was intensified, resulting in a rapid increase in the 4-NP reduction rate.

### 2.4 Component of bicomponent nanocrystals: Au–Ag nanocrystal or Ag_2_S–Ag nanocrystals

**Fig. 5:**
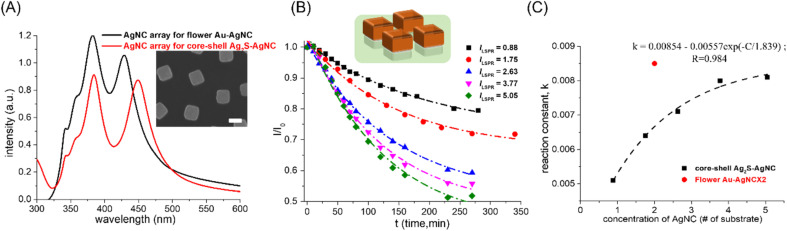
Changes in the reactivity of 4-NP reduction with different concentrations of Ag_2_S–AgNC nanocrystals as catalysts. (A) Extinction spectra of the AgNC array in a polystyrene matrix, which was used to fabricate flower-like Au–AgNCs (black curve) and Ag_2_S–AgNCs (red curve). SEM image of cage-like Au–Ag nanocrystals as an inset image. (B)The concentration of 4-NP varied with reaction time at different nanocrystal concentrations. The dashed line was generated by fitting the curve with an exponential equation. (C) the rate of the 4-NP reduction reaction (reaction constant) varied with the concentration of Au–AgNCs (*C*, # of substrates, Au–AgNC array in a polymer matrix). *k* exhibited an exponential saturation relationship with the increase of *C*. The reaction rate of 4-NP reduction was compared with flower-like Au–AgNCs (blue triangle) and cage-like Au–AgNC nanocrystals (red circle). The scale bar was 100 nm.

Besides noble metals that can be used as 4-NP reduction catalysts, Ag_2_S had also been demonstrated to be an excellent 4-NP reduction catalyst.^14,15^ Here, Ag_2_S–AgNC nanocrystals were synthesized by a sulfidation reaction on the AgNC surface.^22^ The core–shell Ag_2_S–AgNC nanocrystals were used as 4-NP reduction catalysts and the catalytic performance was compared with that of Au–AgNC nanocrystals. The concentration of core–shell Ag_2_S–AgNC nanocrystals can also be characterized by measuring the intensity of the extinction spectrum of the AgNC array on a polystyrene matrix for the synthesis of core–shell Ag_2_S–AgNC nanocrystals (as shown in the red curve in Fig. 5(A)), and the concentration needed to be adjusted by comparing the intensity of the extinction spectrum of the AgNC array on a polystyrene matrix for the synthesis of flower-like Ag–AgNC nanocrystals (as shown in the black curve in Fig. 5(A)). The result showed that the concentration of core–shell Ag_2_S–AgNC nanocrystals (# of Ag_2_S–AgNC) on one substrate was close to 0.88 times that of flower-like Au–AgNC nanocrystals on one substrate. Therefore, the adjusted Ag_2_S–AgNC nanocrystal concentration (# of the substrate) for 4-NP reduction was equal to 0.88 times the actual number of substrates used for 4-NP reduction. The kinetic mechanism of Ag_2_S–AgNC nanocrystals as 4-NP reduction catalysts was also the first-order reaction over a wide range of nanocrystal concentrations as shown in Fig. 5(B) and ESI S6 and Table S5.† However, the reduction rate (reaction constant, *K*) of 4-NP showed an exponential growth and saturation relationship with the catalyst concentration. And the reduction rate of 4-NP with core–shell Ag_2_S–AgNC nanocrystals as catalysts was much lower than that with flower-like Au–AgNC nanocrystals as catalysts (red circle dot in Fig. 5(C)), which may be attributed to the reduction of Ag^+^ ions in Ag_2_S to Ag atoms assisted by Ag and NaBH_4_. The reduction of Ag^+^ ions in Ag_2_S to Ag atoms was a competitive reaction with the reduction of 4-NP, which led to a decrease in the reduction rate of 4-NP. The core–shell Ag_2_S–AgNC nanocrystals underwent the shape change during the 4-NP reduction (as shown in the SEM image in ESI S8(A)†), which can be used as evidence for the reduction of Ag^+^ ions in Ag_2_S to Ag atoms. And the rounded core–shell Ag_2_S–AgNC nanocrystals as 4-NP reduction catalysts showed a faster 4-NP reduction rate than core–shell Ag_2_S–AgNC nanocrystals as 4-NP reduction catalysts, as shown in ESI S8(B) and (C),† which can be used to demonstrate the reduction of Ag_2_S content in Ag_2_S–AgNC nanocrystals by reducing Ag^+^ ions in Ag_2_S to Ag atoms and enhanced the 4-NP reduction reaction (the 4NP reduction rate of pristine AgNCs as catalyst was higher than that of Ag_2_S–AgNCs as catalyst).

The reduction of Ag^+^ ions in Ag_2_S to Ag atoms assisted by NaBH_4_ can be used to explain the exponential growth and saturation relationship between the catalyst concentration and the reduction rate of 4-NP. With the increase in the core–shell Ag_2_S–AgNC nanocrystal concentration, the reduction reaction of Ag^+^ ions in Ag_2_S to Ag atoms was enhanced and the reduction reaction of 4-NP was weakened. The decreases in the reduction rate of 4-PN due to the reduction of Ag^+^ ions in Ag_2_S can be exacerbated by increasing the Ag_2_S contents in core–shell Ag_2_S–AgNC (shown as ESI S9†), which resulted from the more Ag_2_S in core–shell Ag_2_S–AgNCs; the less NaBH_4_ was available for 4-NP reduction.

### 2.5 Irradiation assistance

**Fig. 6:**
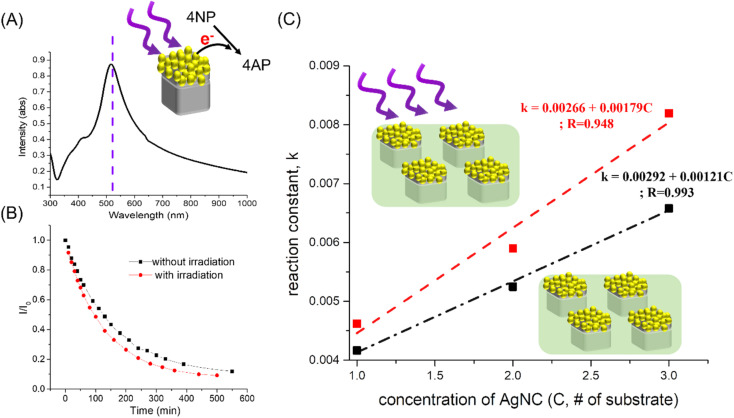
Reactivity of 4-NP reduction with irradiation assistance. (A) Extinction spectrum of the flower-like Au–AgNC array in a polystyrene matrix, showing strong plasmonic mode at 520 nm. (B)The concentration of 4-NP varied with reaction time without (black points) and with (red points) irradiation assistance. The dashed line was generated by fitting the curve with an exponential equation. (C) the rate of the 4-NP reduction reaction (reaction constant) varied with the concentration of Au–AgNCs (*C*, # of substrates, Au–AgNC array in a polymer matrix). *k* exhibited a linear relationship with the increase of *C*. And the enhancement factor of the reaction rate increased with the number of hot electrons (the number of catalysts) under irradiation assistance.

Due to the excellent plasmon properties of Ag nanocrystals, the catalytic performance of Ag-based nanocrystals as 4-NP reduction catalysts should be effectively enhanced under irradiation excitation, which resulted from the generation of hot electrons around Ag-based nanocrystals. Here, the hot electrons were generated by flower-like Au–AgNC nanocrystals at an irradiation wavelength of 520 nm (corresponding to the plasmonic mode of flower-like Au–AgNC nanocrystals shown in Fig. 6(A)) and used to reduce 4-NP. Compared with the flower-like Au–Ag nanocrystals without irradiation assistance (without hot electrons), the flower-like Au–Ag nanocrystals with irradiation assistance have a faster reaction rate of 4-NP reduction, as shown in Fig. 6(B). And the enhancement factor (reaction rate (*k*) with irradiation assistance/reaction rate without irradiation assistance) increased with the number of flower-like Au–Ag nanocrystals as shown in Fig. 6(C) and ESI Table S7.† This phenomenon is due to the increase in the number of hot electrons for enhancing the reduction reaction of 4-NP with the increase of catalyst concentration under the same irradiation power.

Here, Ag-based nanocrystals were used as catalysts for the 4-NP reduction reaction. The reusability of the catalyst can be improved by fixing the catalyst in the polymer matrix. The catalytic performance of these Ag-based nanocrystals strongly depended on their morphologies and components, which are caused by the surface area/volume and synergistic effects, respectively. Since the catalytic activity strongly depended on the amount of catalyst and the concentration-reducing agent (NaBH_4_) and inconsistencies in the use of these parameters for catalytic activity studies in previous reports, it is difficult to demonstrate the catalytic performance directly using the reaction rate (reaction time). Here, the ratio of the amount of catalyst to the amount of 4-NP in aqueous solution and the reaction completion time was used to represent the catalytic performance and comparison with different literature studies, as shown in the ESI Tables S8 and S12.† The catalytic performance of the flower-like Au–Ag nanocrystals indicated that a smaller amount of nanocrystals can be used to degrade the same concentration of 4-NP within the same reaction time period than catalysts in the literature.

These Ag-based nanocrystals with special morphology and composition on a PS matrix used as 4-NP reduction catalysts showed higher catalytic performance (at least two orders of magnitude fewer nanocrystal numbers than in the literature can be used to treat the same concentration of 4-NP in aqueous solution, as shown in ESI S11†).^25^ The catalytic performance of Ag-based nanocrystals can be further enhanced by generating hot electrons around the nanocrystals under irradiation. These results pave the way for designing excellent catalysts with multiple enhancement effects, such as surface area/volume, synergistic effect, and hot electron assisted effect.

## 3. Conclusions

Silver-based nanocrystals have excellent catalytic performance in various reactions, such as 4-PN reduction. Here, bimetallic nanocrystals (Au–Ag) and metal–semiconductor (Ag–Ag_2_S) nanocrystals with highly heterogeneous morphologies, which can be fabricated by a seed-mediated method in the heterogeneous interface (polymer film), act as catalysts for the reduction of 4-NP. Since the heterogeneous nanocrystals are fixed in the polymer film, the reusability of the catalysts is significantly improved compared to the catalysts that are freely dispersed in the reaction solution. These heterogeneous nanocrystals have different morphologies and compositions, leading to different surface area/volume ratios and synergistic effects, resulting in different catalytic performances. The flower-like Au–Ag nanocrystals with higher surface area/volume ratio and cage-like Au–Ag nanocrystals with stronger synergistic effects show faster 4-NP reduction than pristine Ag nanocrystals. And the reduction of Ag_2_S to Ag atom is a competitive reaction with the reduction of 4-NP, resulting in a slower reaction rate of Ag_2_S–Ag nanocrystals than pristine Ag nanocrystals. The catalytic performance of Ag-based nanocrystals can be further enhanced by generating hot electrons around nanocrystals under irradiation. These results show that by carefully controlling the morphology and composition of nanocrystals, it is possible to design and fabricate excellent catalysts for various reactions.

## 4. Experimental section

### 4.1 Materials

Silver nitrate (≥99%, Fluka), 1,5-pentanediol (98%, ACROS), polyvinylpyrrolidone (PVP, *M*_w_ = 55 000 (g mol^−1^), Sigma), copper(ii) chloride (99%, Sigma), sulfur powder (99.5%, ACROS), sodium sulfide nonahydrate (Na_2_S. 9H_2_O, 98%, ACROS), polystyrene (PS, *M*_w_ = 35 000 (g mol^−1^), Sigma), hydrogen tetrachloroaurate(iii) trihydrate (99.99%, Alfa Aesar), 4-nitrophenol (99%, Alfa Aesar), sodium borohydride, (NaBH_4_, 99%, ACROS), ethanol (95%), toluene (>99.5%, J. T. Baker), and chloroform (99.8%, Macron) were used.

### 4.2 Synthesis of shaped AgNCs

AgNCs were synthesized by using the polyol synthetic method which has been reported in previous literature. The AgNO_3_ precursor solution was prepared by dissolving 0.20 g of AgNO_3_ in 5 mL of 1,5-pentanediol with 44 μL of 0.043 M CuCl_2_ solution. Another precursor solution was prepared by dissolving 0.10 g of PVP in 5 mL of 1,5-pentanediol. The reaction solution was prepared by heating 10 mL of 1,5-pentanediol in a 50 mL glass round-bottom flask with continuous stirring in an oil bath heated to 193 °C. AgNO_3_ and PVP precursor solutions were injected alternately into the hot reaction solution at rates of 500 μL of AgNO_3_ precursor solutions per min and 320 μL of PVP precursor solutions per 30 s. Some reaction parameters, such as the total volume of precursor injection in the reaction batch and reaction temperature, can be used to control the size of AgNCs. To obtain highly uniform heterogeneous nanocrystals through the seed-mediated synthesis method, the quality (shape and size) of AgNCs (seeds) needs to be enhanced, which can be achieved by a post-synthetic purification process. The as-made AgNC colloidal solution contained ∼20% impurities of other shapes, such as triangular, spherical, and wire-like nanocrystals, and the uniformity of AgNCs could be increased to >95% after four times of filtration with 450 nm filter paper and six times with 220 nm filter paper.

### 4.3 Deposition of polystyrene thin films on the substrate

There are three steps for the deposition of PS films on silicon/glass substrates: (1) clean silicon/glass substrates with a freshly prepared piranha solution (70% (v) concentrated H_2_SO_4_ and 30% (v) H_2_O_2_); (2) formation of a hydrophobic coating on the substrate by treated substrates with hexamethyldisilazane (HMDS) vapor, which can be used to enhance the adhesion force between the substrate and the polymer thin film; (3) deposition of the PS film on substrates; PS (*M*_w_ = 35 000) was dissolved in a toluene solution (5.8 wt%) for deposition of thin films on the substrate *via* the spin-coating process. The thickness of PS films on substrates can be controlled by the concentration of PS solution and spin speed.

### 4.4 Deposition of AgNC arrays on PS films

To obtain AgNC arrays on PS films, the as-synthesized and purified AgNC colloidal solutions were precipitated in ethanol and then dispersed in CHCl_3_. Then, the colloidal solution of AgNCs/CHCl_3_ was added dropwise to the water–air interface of a glass Petri dish with water, and CHCl_3_ leaving volatilized to leave a monolayer of silver nanocrystals with isotopically distributed floating on the water–air interface. The surface tension of the AgNC monolayer array at the water–air interface can be used to control the concentration (surface coverage) of the AgNC array. The AgNC monolayer array was then transferred onto the PS films by dip-coating. The AgNC array on the PS film can then be embedded into the PS film by thermal treatment at a temperature above the glass-transition temperature of PS (*T* > *T*_g of PS_). The embedding depth of AgNCs in the PS film can be controlled by thermal treatment conditions (treatment temperature and time).

### 4.5 Using AgNCs partially embedded in the polymer matrix as a template to synthesize flower-like Au–AgNC nanocrystals

The AgNC was partially embedded into PS film by thermal treatment, and its embedding depth (*H*) was about *H* ∼ 2*L*/3 by (*L* is the length of AgNCs). This AgNC-PS-glass or silicon substrate was hung in an aqueous solution (as shown in the schematic in ESI S13†) for the synthesis of bi-component nanocrystals. Dissolving 8 mg of PVP in a 20 ml glass vial with 12 ml of DI water (0.012 mM) as a reaction solution. Under magnetic stirring, 11.2 μl of 1 mM HAuCl_4_ solution was injected into the reaction solution every 20 minutes, and Au–Ag bimetallic nanocrystals were formed by reducing Au^3+^ ions. The change of optical properties with reaction time can be characterized by measuring the extinction spectrum of the AgNC-PS-glass substrate. And the shape change of Au–Ag alloy nanocrystals with time can be observed by SEM and HRTEM.^21^

### 4.6 Using AgNCs partially embedded in the polymer matrix as a template to synthesize cage-like Au–AgNC nanocrystals

The AgNC was partially embedded into the PS film by thermal treatment, and its embedding depth (*H*) was about *H* ∼ 2*L*/3 (*L* is the length of AgNCs). This AgNC-PS-glass or silicon substrate was hung in an aqueous solution for the synthesis of bi-component nanocrystals. Dissolving 1 mg of PVP in a 20 ml glass vial with 12 ml of DI water (0.0015 mM) gave the reaction solution. Under magnetic stirring, 11.2 μl of 1 mM HAuCl_4_ solution was injected into the reaction solution every 20 minutes, and Au–Ag bimetallic nanocrystals were formed by reducing Au^3+^ ions. The change of optical properties with reaction time can be characterized by measuring the extinction spectrum of the AgNC-PS-glass substrate. And the shape change of Au–Ag alloy nanocrystals with time can be observed by SEM and HRTEM.^21^

### 4.7 Using AgNCs partially embedded in the polymer matrix as a template to synthesize core–shell-like Ag_2_S–Ag nanocrystals.

The AgNC was partially embedded into the PS film by thermal treatment, and its embedding depth (*H*) was about *H* ∼ *L*/3 by (*L* is the length of AgNCs) (as ESI S1†). This AgNC-PS-glass or silicon substrate was hung in an aqueous solution for the synthesis of bi-component nanocrystals. Dissolving 12 mg of PVP in a 20 ml glass vial with 18 ml of DI water (0.018 mM) as a reaction solution. Two reaction precursor solutions were prepared by dissolving 16.4 mg of S powder and 72.2 mg of Na_2_S–9H_2_O in 6.3 ml of DI water at 80 °C with magnetic stirring for 12 h (forming the 0.047 mM Na_2_S_*x*_ solution). The color of the Na_2_S_*x*_ aqueous solution is bright yellow. And dilute the Na_2_S_*x*_ aqueous solution to 0.0004 mM (10 μL of 0.047 mM Na_2_S_*x*_ aqueous solution + 1.2 mL DI H_2_O). Under magnetic stirring, 10 μl of 0.0004 mM Na_2_S_*x*_ solution was injected into the reaction solution every 20 minutes, and Ag_2_S–Ag bicomponent nanocrystals were formed. The change of optical properties with reaction time can be characterized by measuring the extinction spectrum of the AgNC-PS-glass substrate. And the shape change of Au–Ag alloy nanocrystals with time can be observed by SEM and HRTEM.^22^

### 4.8 Catalytic performance test of silver-based nanocrystals fixed on a PS matrix as catalysts for the reduction of 4-nitrophenol

The as-made Ag-based nanocrystals on the PS matrix were mounted on a plastic plate (as shown in the schematic in ESI S10†) and placed in a quartz cuvette. 1.7 mM 4-NP aqueous solution (2.087 mg 4-NP dissolved in 8.82 ml DI water) and 0.4 M NaBH_4_ aqueous solution (38 mg NaBH_4_ dissolved in 2.5 ml DI water) were prepared for 4-NP reduction test. For the 4-NP reduction test, 2.5 ml DI water and 150 μl 1.7 mM 4-NP aqueous solution were added to a quartz cuvette with an Ag-based nanocrystal-PS matrix. And then 150 μl 0.4 M NaBH_4_ aqueous solution was injected into the quartz cuvette (reaction time = 0 min). The color of the aqueous solution immediately changed from colorless to yellow (nitrophenolate ions). Then, the variation of the absorption peak intensity of nitrophenolate ions (∼400 nm) with reaction time was used to observe the reduction rate of 4-NP.

### 4.9 Catalytic performance test of freely dispersed silver-based nanocrystals as catalysts for the reduction of 4-nitrophenol

The as-made Ag-based nanocrystals on the PS matrix were placed in chloroform to remove Ag-based nanocrystals from the PS matrix. And Ag-based nanocrystals were then dispersed in DI water. 1.7 mM 4-NP aqueous solution (2.087 mg 4-NP dissolved in 8.82 ml DI water) and 0.4 M NaBH_4_ aqueous solution (38 mg NaBH_4_ dissolved in 2.5 ml DI water) were prepared for the 4-NP reduction test. For the 4-NP reduction test, 2.6 ml DI water and 150 ml 1.7 mM 4-NP aqueous solution were added to a quartz cuvette with free dispersed Ag-based nanocrystals. And then 150 ml of 0.4 mM NaBH_4_ aqueous solution was injected into the quartz cuvette (reaction time = 0 min). The color of the aqueous solution immediately changed from colorless to yellow (nitrophenolate ions). Then, the variation of the absorption peak intensity of nitrophenolate ions (∼400 nm) with reaction time was used to observe the reduction rate of 4-NP.

### 4.10 Catalytic performance test of flower-like Au–AgNC nanocrystals fixed on a PS matrix as photocatalysts for the reduction of 4-nitrophenol

The as-made flower-like Au–AgNC nanocrystals on the PS matrix were mounted on a plastic plate (as shown in the schematic in ESI S10†) and placed in a quartz cuvette. 1.7 mM 4-NP aqueous solution (2.087 mg 4-NP dissolved in 8.82 ml DI water) and 0.4 M NaBH_4_ aqueous solution (38 mg NaBH_4_ dissolved in 2.5 ml DI water) were prepared for 4-NP reduction test. For the 4-NP reduction test, 2.5 ml DI water and 150 μl 1.7 mM 4-NP aqueous solution were added to a quartz cuvette with an Ag-based nanocrystal-PS matrix. And then 150 μl of 0.4 M NaBH_4_ aqueous solution was injected into the quartz cuvette (reaction time = 0 min). The color of the aqueous solution immediately changed from colorless to yellow (nitrophenolate ions). The external light source is an LED point light source with a wavelength of ∼520 nm and power ∼9.9 W which was used to generate the “hot electrons” on nanocrystals. The spot size of the external light source on the nanocomposite sample is about 1 cm in diameter (sample size 1 cm × 1 cm square), which can be adjusted by changing the distance between the light source and the sample. The power density of irradiation is ∼12.6 W cm^−2^. Then, the variation of the absorption peak intensity of nitrophenolate ions (∼400 nm) with reaction time was used to observe the reduction rate of 4-NP.

## Author contributions

Su-Wen Hsu conceived the idea. Ming-Shiuan Huang, Hsien-Tai Cheng, and Su-Wen Hsu designed the research. Ming-Shiuan Huang and Hsien-Tai Cheng analyzed the data. The manuscript was written and approved by all authors.

## Conflicts of interest

The authors declare no competing financial interest.

## Supplementary Material

NA-005-D3NA00473B-s001

## References

[cit1] Liao G. F., Fang J. S., Li Q., Li S. H., Xu Z. S., Fang B. Z. (2019). Ag-Based nanocomposites: synthesis and applications in catalysis. Nanoscale.

[cit2] Mika L. T., Csefalvay E., Nemeth A. (2018). Catalytic Conversion of Carbohydrates to Initial Platform Chemicals: Chemistry and Sustainability. Chem. Rev..

[cit3] Ai Y. J., Hu Z. N., Shao Z. X., Qi L., Liu L., Zhou J. J., Sun H. B., Liang Q. L. (2018). Egg-like magnetically immobilized nanospheres: A long-lived catalyst model for the hydrogen transfer reaction in a continuous-flow reactor. Nano Res..

[cit4] Gao D. W., Zhang X., Dai X. P., Qin Y. C., Duan A. J., Yu Y. B., Zhuo H. Y., Zhao H. R., Zhang P. F., Jiang Y., Li J. M., Zhao Z. (2016). Morphology-selective synthesis of active and durable gold catalysts with high catalytic performance in the reduction of 4-nitrophenol. Nano Res..

[cit5] Saran S., Manjari G., Devipriya S. P. (2018). Synergistic eminently active catalytic and recyclable Ag, Cu and Ag-Cu alloy nanoparticles supported on TiO2 for sustainable and cleaner environmental applications: A phytogenic mediated synthesis. J. Cleaner Prod..

[cit6] Meng L., Liu Z. H., Lan C. W., Xu N. (2022). In-Situ Fabricating Ag Nanoparticles on TiO2 for Unprecedented High Catalytic Activity of 4-Nitrophenol Reduction. Catal. Lett..

[cit7] Zhou J. D., Liu X. L., Huang J. J., Guo D., Sun H., Xu C. Z., Zhang J. Z., Ji X., Ma J. J., Liu L., Tong Z. W. (2022). Construction of novel Ag@SrNbO/LDH ternary hybrid with high catalytic performance towards the reduction of 4-nitrophenol. Appl. Surf. Sci..

[cit8] Gangula A., Podila R., Ramakrishna M., Karanam L., Janardhana C., Rao A. M. (2011). Catalytic Reduction of 4-Nitrophenol using Biogenic Gold and Silver Nanoparticles Derived from Breynia rhamnoides. Langmuir.

[cit9] Akilandaeaswari B., Muthu K. (2021). One-pot green synthesis of Au-Ag bimetallic nanoparticles from Lawsonia inermis seed extract and its catalytic reduction of environmental polluted methyl orange and 4-nitrophenol. J. Taiwan Inst. Chem. Eng..

[cit10] Princy K. F., Gopinath A. (2021). Green synthesis of silver nanoparticles using polar seaweed Fucus gardeneri and its catalytic efficacy in the reduction of nitrophenol. Polar Sci..

[cit11] Mourdikoudis S., Altantzis T., Liz-Marzan L. M., Bals S., Pastoriza-Santos I., Perez-Juste J. (2016). Hydrophilic Pt nanoflowers: synthesis, crystallographic analysis and catalytic performance. Crystengcomm.

[cit12] Grzeschik R., Schafer D., Holtum T., Kupper S., Hoffmann A., Schlucker S. (2020). On the Overlooked Critical Role of the pH Value on the Kinetics of the 4-Nitrophenol NaBH4-Reduction Catalyzed by Noble-Metal Nanoparticles (Pt, Pd, and Au). J. Phys. Chem. C.

[cit13] Liao G. F., Gong Y., Zhong L., Fang J. S., Zhang L., Xu Z. S., Gao H. Y., Fang B. Z. (2019). Unlocking the door to highly efficient Ag-based nanoparticles catalysts for NaBH4-assisted nitrophenol reduction. Nano Res..

[cit14] Lang B., Yu H. K. (2017). Novel Ag2S nanoparticles on reduced graphene oxide sheets as a super-efficient catalyst for the reduction of 4-nitrophenol. Chin. Chem. Lett..

[cit15] Dutta D., Hazarika R., Dutta P. D., Goswami T., Sengupta P., Dutta D. K. (2016). Synthesis of Ag-Ag2S Janus nanoparticles supported on an environmentally benign cellulose template and their catalytic applications. RSC Adv..

[cit16] Das T. K., Das N. C. (2022). Advances on catalytic reduction of 4-nitrophenol by nanostructured materials as benchmark reaction. Int. Nano Lett..

[cit17] Das T. K., Ghosh S. K., Das N. C. (2023). Green synthesis of a reduced graphene oxide/silver nanoparticles-based catalyst for degradation of a wide range of organic pollutants. Nano-Struct. Nano-Objects.

[cit18] Jeong J. H., Pradyast A., Shim H., Woo H. C., Kim M. H. (2021). Completely green synthesis of rose-shaped Au nanostructures and their catalytic applications. RSC Adv..

[cit19] Kastner C., Thunemann A. F. (2016). Catalytic Reduction of 4-Nitrophenol Using Silver Nanoparticles with Adjustable Activity. Langmuir.

[cit20] Tolaymat T. M., El Badawy A. M., Genaidy A., Scheckel K. G., Luxton T. P., Suidan M. (2010). An evidence-based environmental perspective of manufactured silver nanoparticle in syntheses and applications: A systematic review and critical appraisal of peer-reviewed scientific papers. Sci. Total Environ..

[cit21] Jhang W. L., Huang M. S., Hsu S. W. (2021). Unsymmetrical Heterogeneous Au-Ag Nanocrystals as Catalysts, Sensors, and Drug Carriers. ACS Appl. Nano Mater..

[cit22] Cheng H. T., Huang M. S., Hsu S. W. (2022). Combination of Plasmon-Mediated Photochemistry and Seed-Mediated Methods for Synthesis of Bicomponent Nanocrystals. ACS Omega.

[cit23] Wunder S., Polzer F., Lu Y., Mei Y., Ballauff M. (2010). Kinetic Analysis of Catalytic Reduction of 4-Nitrophenol by Metallic Nanoparticles Immobilized in Spherical Polyelectrolyte Brushes. J. Phys. Chem. C.

[cit24] Mei Y., Sharma G., Lu Y., Ballauff M., Drechsler M., Irrgang T., Kempe R. (2005). High catalytic activity of platinum nanoparticles immobilized on spherical polyelectrolyte brushes. Langmuir.

[cit25] Holden M. S., Nick K. E., Hall M., Milligan J. R., Chen Q., Perry C. C. (2014). Synthesis and catalytic activity of pluronic stabilized silver-gold bimetallic nanoparticles. RSC Adv..

