# Supplementary material for: Effect of morphologies and compositions of silver-based multicomponent heterogeneous nanocrystals on the reduction of 4-nitrophenol
Source: Nanoscale Adv. 2023 Aug 30;5(18):4968–78. doi: 10.1039/d3na00473b (PMC10496911; doi:10.1039/d3na00473b)
Supplement: NA-005-D3NA00473B-s001 [file NA-005-D3NA00473B-s001.pdf]

Electronic Supplementary Material (ESI) for Nanoscale Advances.
This journal is © The Royal Society of Chemistry 2023

Table S1 Stability testing of flower Au-AgNC nanocrystal as catalysts: curves fitted with exponential decay equations.

Concentration of flower-like AgNC	**Fitting formula：$I=I_0+A*exp(-(t-t_0)/B)$; B = 1/ reaction constant (K)**					
	y_0	t_0	A	B	K=1/B	R-square
Free catalysts (Flower Au-AgNC)						
1st cycle rxn	-0.471	6.938	1.272	35.29	0.0283	0.953
1st cycle rxn	-0.0146	2.793	1.052	71.246	0.0140	0.975
1st cycle rxn	115.920	4.45×10^6	-42.86	4.51×10^6	~0	0.410
Fixed catalysts (Flower Au-AgNC)						
1st cycle rxn	0.0313	-1.623	0.953	88.15	0.0113	0.997
1st cycle rxn	0.0202	-0.324	0.978	83.39	0.0120	0.999
1st cycle rxn	0.0202	-0.324	0.979	83.26	0.0120	0.999

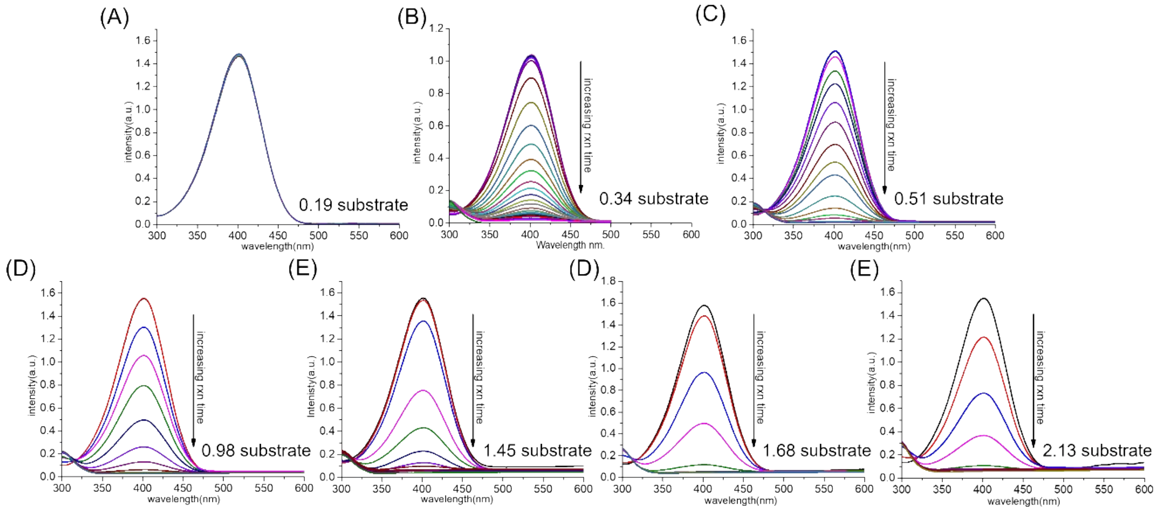

S1 Concentration-dependent reactivity tests of flower Au-AgNC nanocrystals as catalysts freely suspended in the reaction solution were characterized by the change of 4-NP concentration in extinction spectra with reaction time. To compare with the catalytic performance of flower Au-AgNC nanocrystals fixed in a polystyrene matrix, the concentration of flower Au-AgNC nanocrystals freely suspended in the reaction solution was converted into the amount of substrate, which had flower Au-AgNC nanocrystals were fixed on it.

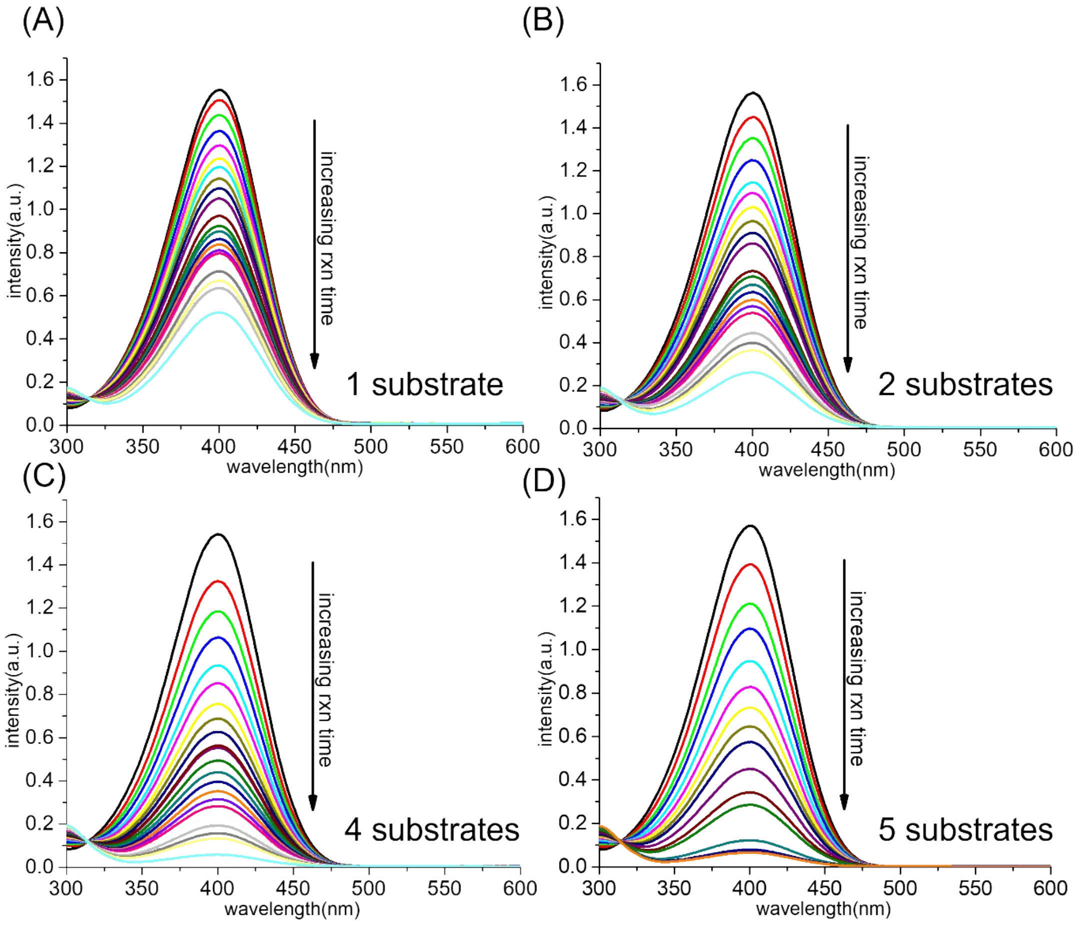

S2 Concentration-dependent reactivity tests of flower Au-AgNC nanocrystals as catalysts fixed in a polystyrene matrix were characterized by the change of 4-NP concentration in extinction spectra with reaction time. The concentration of flower Au-AgNC nanocrystals fixed in a polystyrene matrix was measured by the amount of the substrate.

Table S2 Concentration-dependent reactivity tests of flower Au-AgNC nanocrystal as catalysts freely suspended in reaction solution: curves fitted with exponential decay equations.

Concentration of free flower Au-AgNC (# of substrate)	Fitting formula：$y=y_0+A*exp(-(t-t_0)/B)$; B = 1/ reaction constant (K)					
	Y_0	t_0	A	B	K=1/B	R-square
I_{LSPR} = 0.19	1.0616	49.79	-0.024	285.66	0.0035	0.878
I_{LSPR} = 0.34	-0.0057	71.56	1.021	71.246	0.014	0.996
I_{LSPR} = 0.51	-0.0389	24.00	1.016	64.526	0.0155	0.980
I_{LSPR} = 0.98	-0.03184	12.00	1.038	36.208	0.02762	0.961
I_{LSPR} = 1.45	0.00218	10.99	1.002	24.506	0.04063	0.972
I_{LSPR} = 1.68	0.02669	10.18	0.922	18.514	0.05402	0.989
I_{LSPR} = 2.13	0.02028	0.146	0.989	17.600	0.05682	0.992

Table S3 Concentration-dependent reactivity tests of flower Au-AgNC nanocrystal as catalysts fixed in a polystyrene matrix: curves fitted with exponential decay equations.

Concentration of fixed flower-Au-AgNC (# of substrate)	Fitting formula：$y=y_0+A*exp(-(t-t_0)/B)$; B = 1/ reaction constant (K)					
	y_0	t_0	A	B	K=1/B	R-square
I_{LSPR} = 1	0.2774	4.302	0.716	139.17	0.0072	0.982
I_{LSPR} = 2	0.1195	1.332	0.872	117.51	0.0085	0.996
I_{LSPR} = 4	0.0313	-1.623	0.953	88.15	0.0113	0.961
I_{LSPR} = 5	0.0150	0.067	0.988	77.12	0.0130	0.972

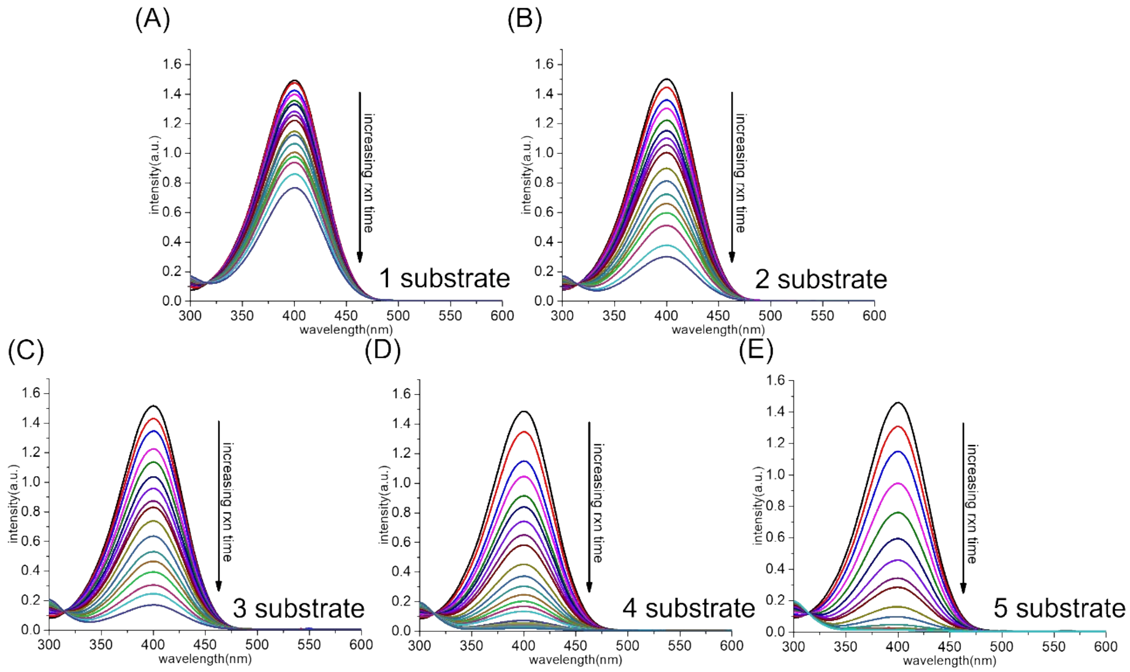

S3 Concentration-dependent reactivity tests of cage Au-AgNC nanocrystals as catalysts fixed in a polystyrene matrix were characterized by the change of 4-NP concentration in extinction spectra with reaction time. The concentration of cage Au-AgNC nanocrystals fixed in a polystyrene matrix was measured by the amount of the substrate.

Table S4 Concentration-dependent reactivity tests of cage Au-AgNC nanocrystal as catalysts fixed in a polystyrene matrix: curves fitted with exponential decay equations.

Concentration of fixed cage- Au-AgNC (# of substrate)	**Fitting formula： $y=y_0+A*exp(-(t-t_0)/B)$; B = 1/ reaction constant (K)**					
	y_0	t_0	A	B	K=1/B	R-square
$I_{LSPR} = 1$	0.4444	2.781	0.560	191.33	0.0052	0.994
$I_{LSPR} = 2$	0.1104	-0.199	0.901	163.28	0.0061	0.998
$I_{LSPR} = 3$	0.0803	0.600	0.933	114.92	0.0087	0.998
$I_{LSPR} = 4$	0.0116	-0.229	0.990	84.42	0.0118	0.999
$I_{LSPR} = 5$	-0.0420	2.629	1.053	55.69	0.0180	0.988

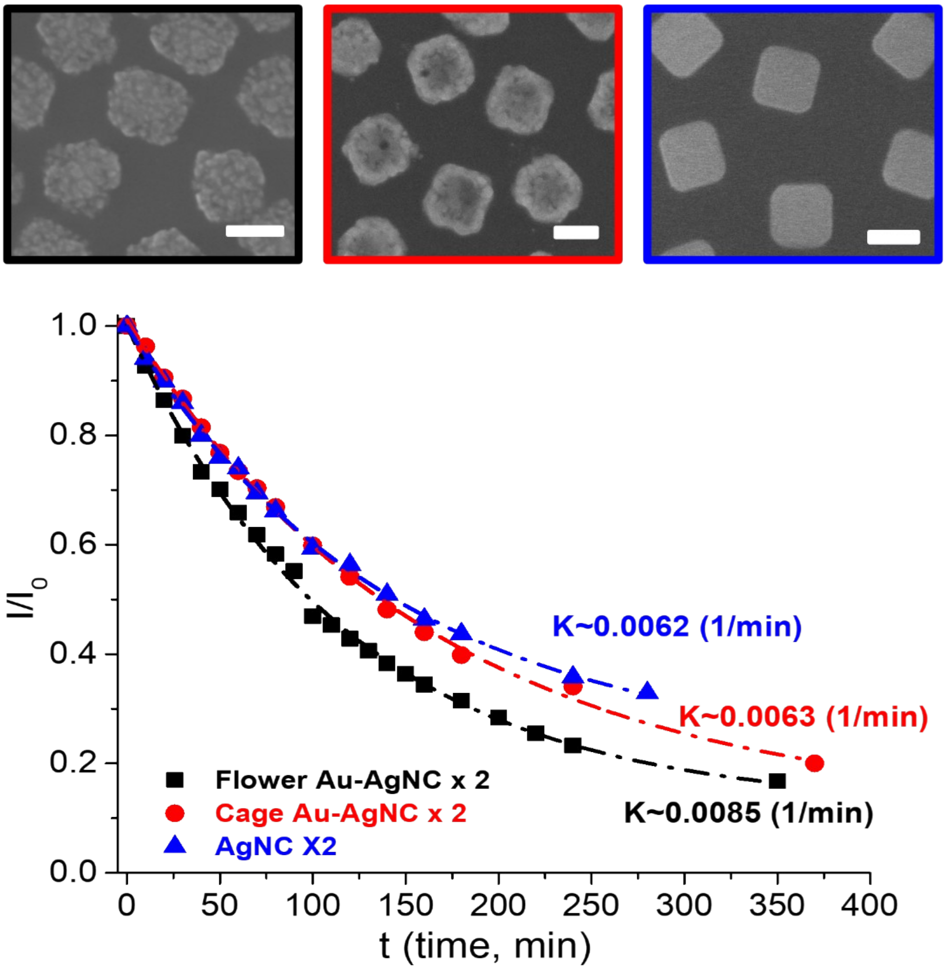

S4 Shape-dependent reactivity tests of silver-based nanocrystals as catalysts fixed in a polystyrene matrix. SEM images showed three different morphologies of silver-based nanocrystals: flower Au-AgNC (black), cage Au-AgNC (red), and pristine AgNC(blue). Changes in the concentration of 4-NP with three different morphologies nanocrystal as catalysts decayed exponentially with reaction time. The scale bar was 100 nm.

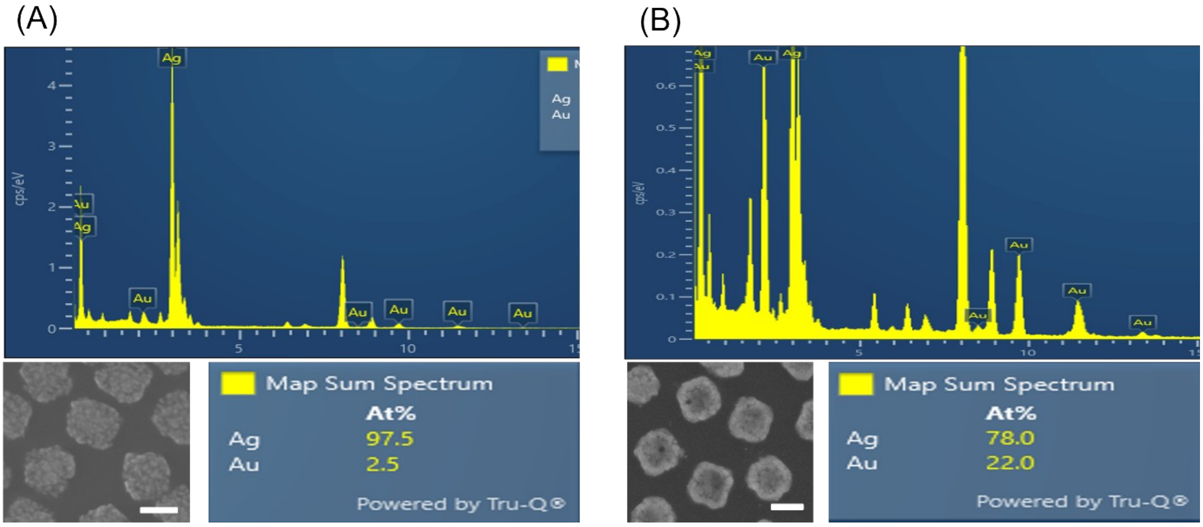

S5 Energy-dispersive X-ray spectroscopy (EDS) of flower Au-AgNC nanocrystal (A) and cage Au-AgNC nanocrystal (B) exhibited that the gold content in flower Au-AgNC nanocrystal was 2.5 at% and the gold content in cage Au-AgNC nanocrystal was 22.0 at%.

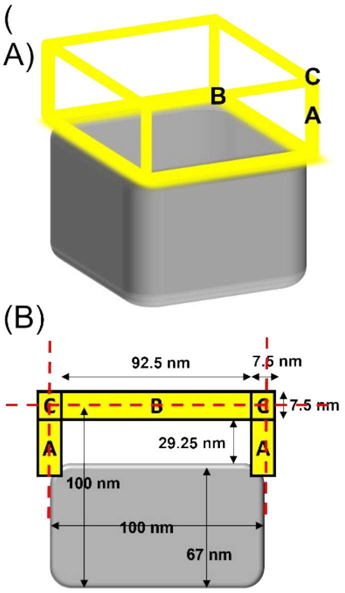

(C) **Length of AgNC = 100 nm   Embedded depth of AgNC = 67 nm**

width of cage Au-AgNC = 7.5 nm

Surface area of A : 7.5 x (33 - 7.5/2) nm^2

Surface area of B : 7.5 x (100 - 7.5) nm^2

Surface area of C : 7.5 x 7.5 nm^2

# of A in half-cage Au-AgNC : 4 x 4

# of B in half-cage Au-AgNC : 4 x 4

# of C in half-cage Au-AgNC : (6-3) x 4

(D) **Total surface area in half-cage Au-AgNC**
= 4 x 4 x 7.5 x 29.25 + 4 x 4 x 7.5 x 92.5 + 3 x 4 x 7.5 x 7.5 = 14835 nm^2

Total surface area in embedded AgNC
= 4 x 33 x 100 + 100 x 100= 23200 nm^2

S6 Theoretical calculation of the surface area of cage-like Au-AgNC and pristine AgNC as 4-NP reduction catalysts. (A) schematic of cage-like Au-AgNC. (B) schematic cross-section of cage-like Au-AgNC. (C) theoretical calculation of the surface area of cage-like Au-AgNC. (D) Theoretical calculation of the surface area of pristine AgNC.

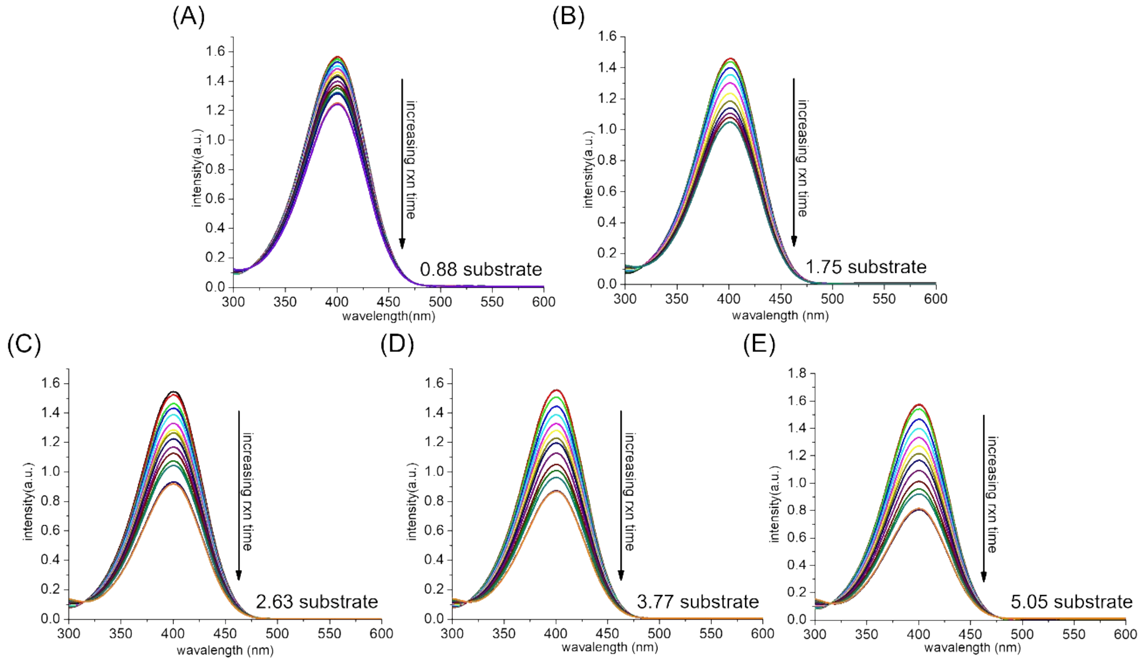

S7 Concentration-dependent reactivity tests of Ag_2S-AgNC nanocrystals as catalysts fixed in a polystyrene matrix were characterized by the change of 4-NP concentration in extinction spectra with reaction time. The concentration of cage Ag_2S -AgNC nanocrystals fixed in a polystyrene matrix was measured by the amount of the substrate, which was adjusted by the number of nanocrystals in a polystyrene matrix.

Table S5 Concentration-dependent reactivity tests of Ag_2S-AgNC nanocrystal as catalysts fixed in a polystyrene matrix: curves fitted with exponential decay equations.

Concentration of fixed core shell-Ag_2S- AgNC (# of substrate)	**Fitting formula：$y=y_0+A*exp(-(t-t_0)/B)$; B = 1/ reaction constant (K)**					
	y_0	t_0	A	B	K=1/B	R-square
$I_{LSPR} = 0.88$	0.7200	16.085	0.270	195.73	0.0051	0.994
$I_{LSPR} = 1.75$	0.6690	10.878	0.326	156.34	0.0064	0.990
$I_{LSPR} = 2.63$	0.5129	9.306	0.468	140.75	0.0071	0.987
$I_{LSPR} = 3.77$	0.4761	9.153	0.519	124.10	0.0080	0.990
$I_{LSPR} = 5.05$	0.4238	22.828	0.517	123.48	0.0081	0.984

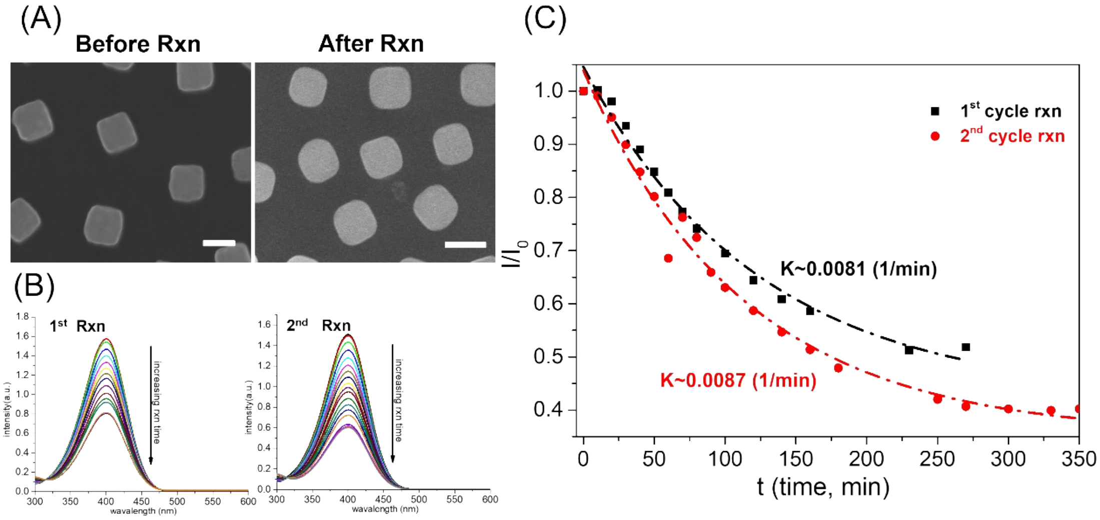

S8 The morphology of Ag_2S-AgNC nanocrystals changed with the reaction as shown in (A). The stability tests of Ag_2S-AgNC nanocrystals fixed as catalysts in a polystyrene matrix were characterized by the change in extinction spectra with reaction time as shown in (B). (C) Changes in the concentration of 4-NP during 1st cycle (black point) and 2nd cycle (red points) reaction decayed exponentially with reaction time.

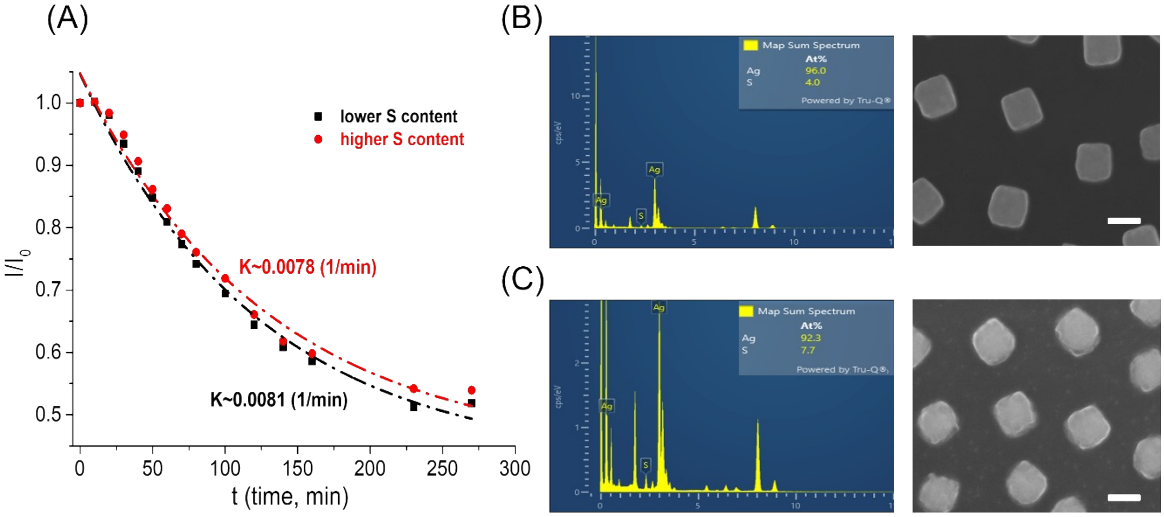

S9 Changes in the concentration of 4-NP under Ag2S-AgNC nanocrystals with different S contents as catalysts decayed exponentially with reaction time as shown in (A). There were two different sulfur contents in Ag_2S-AgNC nanocrystal as catalysts: the sulfur content in Ag_2S-AgNC nanocrystal was 4.0 at% as EDX shown in (B). And the sulfur content in Ag_2S -AgNC nanocrystal was 7.7 at% as EDX shown in (C).

Table S6 Stability test and sulfur content-dependent reactivity tests of Ag_2S-AgNC nanocrystal as catalysts fixed in a polystyrene matrix: curves fitted with exponential decay equations

Concentration of fixed core shell-Ag_2S- AgNC (# of substrate)	**Fitting formula：$y=y_0+A*exp(-(t-t_0)/B)$; B = 1/ reaction constant (K)**					
	y_0	t_0	A	B	K=1/B	R-square
I_{LSPR} = 5.05 1st cycle rxn	0.4238	22.828	0.517	123.48	0.0081	0.984
I_{LSPR} = 5.05 2nd cycle rxn	0.3520	6.147	0.650	113.93	0.0087	0.984

Concentration of fixed core shell-Ag_2S- AgNC (# of substrate)	**Fitting formula：$y=y_0+A*exp(-(t-t_0)/B)$; B = 1/ reaction constant (K)**					
	Y_0	t_0	A	B	K=1/B	R-square
I_{LSPR} = 5.05 lower S content	0.4238	22.828	0.517	123.48	0.0081	0.984
I_{LSPR} = 5.05 higher S content	0.4392	26.114	0.500	128.80	0.0078	0.984

Table S7 Flower-like Au-AgNC nanocrystal as catalysts with and without irradiation assistance

Concentration of fixed flower-Au-AgNC (# of substrate) without irradiation assistance	**Fitting formula：y=y$_0$+A*exp(-(t-t$_0$)/B) ; B = 1/ reaction constant (K)**					
	y_0	t_0	A	B	K=1/B	R-square
$I_{LSPR} = 1$	0.7391	20.9302	0.6656	240.0767	0.0042	0.991
$I_{LSPR} = 2$	0.1462	3.8174	0.8466	190.5623	0.0052	0.999
$I_{LSPR} = 3$	0.0985	0.2222	0.8988	152.0171	0.0066	0.999

Concentration of fixed flower-Au-AgNC (# of substrate) with irradiation assistance	**Fitting formula：y=y$_0$+A*exp(-(t-t$_0$)/B) ; B = 1/ reaction constant (K)**					
	y_0	t_0	A	B	K=1/B	R-square
$I_{LSPR} = 1$	0.5138	5.6058	0.4807	216.5481	0.0046	0.998
$I_{LSPR} = 2$	0.1649	9.8275	0.7920	168.5371	0.0059	0.999
$I_{LSPR} = 3$	0.0761	9.7592	0.8398	123.8766	0.0082	0.999

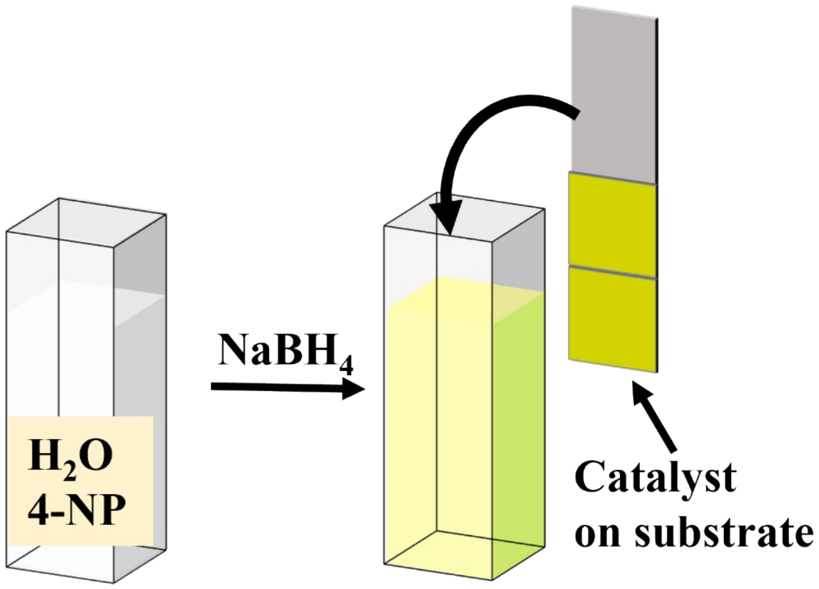

S10 The schematic of reaction equipment setting up which includes the 4-NP aqueous solution in a quartz cuvette. The reducing agent ($NaBH_4$ aqueous solution) and Ag-based nanocrystals-PS matrix were then added into the quartz cuvette to study the reduction rate of 4-NP.

(A) **Reference[1] :**

catalyst (Ag nanoparticle): $1 \times 10^{-9}\ M, 300\ \mu L = 3 \times 10^{-13}$ mole

4-NP concentration: $1.7 \times 10^{-3} M, 0.15$ mL $= 2.25 \times 10^{-7}$ mole

$NaBH_4$ concentration: $0.015M, 1$ mL $= 1.5 \times 10^{-5}$ mole

catalyst :4-NP : $NaBH_4$ = 1.33×10^{-6} : 1 : 66.7

(B) **Intensity of AgNC = 1.0 → surface coverage % of substrate = 21 %;**
substrate size 1cm × 1cm and AgNC size = 100 nm (AgNC project area = 10^4 nm²)

$$\#\ of\ AgNC\ in\ substrate: \frac{10^{14}(nm^2) \times 0.22}{10^4(\frac{nm^2}{AgNC})} = 2.1 \times 10^9$$

$$\frac{2.1 \times 10^9}{6.02 \times 10^{23}(\frac{\#\ of\ AgNC}{mole})} = 3.45 \times 10^{-15}\ mole$$

4-NP concentration: $1.7 \times 10^{-3}\ M, 0.15$ mL $= 2.25 \times 10^{-7}$ mole

$NaBH_4$ concentration: $0.4\ M, 0.15$ mL $= 6 \times 10^{-5}$ mole

catalyst :4-NP : $NaBH_4$ = 1.53×10^{-8} : 1 : 266.7

The amount of catalyst used to treat the same amount of 4NP is 0.0115 times

S11 Comparison of the number of silver nanocrystals in the literature and that used in our experiment when treatment with the same concentration of 4-NP aqueous solution.

Table S8 compared the catalytic performance of Ag-based bicomponent nanocrystals with other literatures.

Catalysts type		# of catalyst (mole)	4-NP (mole)	$NaBH_4$(mole)	# of catalyst /4-NP	Overall rxn time	Reference
Ag based bicomponent NP	Ag-Au Flower (fixe catalyst)	3.45×10^{-15}	2.25×10^{-7}	6×10^{-5}	1.53×10^{-8}	~250 min	This manuscript
	Ag-Au Flower (freely suspended)	2.07×10^{-14}	2.25×10^{-7}	6×10^{-5}	9.20×10^{-8}	~ 3 min	
	Ag-Au cage (fixe catalyst)	3.45×10^{-15}	2.25×10^{-7}	6×10^{-5}	1.53×10^{-8}	~175 min	
	$Ag-Ag_2S$ (fixe catalyst)	3.45×10^{-15}	2.25×10^{-7}	6×10^{-5}	1.53×10^{-8}	~350 min	
Ag-Au bimetallic NP		3×10^{-13}	2.25×10^{-7}	1.5×10^{-5}	1.33×10^{-6}	~ 3 min	23
Ag-Au bimetallic NP + GO		1.8×10^{-8}	3.85×10^{-6}	4.4×10^{-4}	4.67×10^{-2}	~ 5 min	a
Au NP		8.8×10^{-8}	1.35×10^{-6}	2.145×10^{-3}	6.52×10^{-2}	~5 min	b
Ag NP		1.39×10^{-5}	3.0×10^{-7}	3×10^{-5}	0.463	~8 min	c

a. Wu, T.; Zhang, L.; Gao, J.; Liu, Y.; Gao, C.; Yan, J. Fabrication of graphene oxide decorated with Au–Ag alloy nanoparticles and its superior catalytic performance for the reduction of 4-nitrophenol. J. Mater. Chem. A 2013, 1 (25), 7384– 7390.
b. K. Kuroda, T. Ishida, M. Haruta, Reduction of 4-nitrophenol to 4-aminophenol over Au nanoparticles deposited on PMMA, J. Mol. Catal. A Chem., 298 (1) (2009), pp. 7-11.
c. S. Jana, S.K. Ghosh, S. Nath, S. Pande, S. Praharaj, S. Panigrahi, S. Basu, T. Endo, T. Pal, Synthesis of silver nanoshell-coated cationic polystyrene beads: A solid phase catalyst for the reduction of 4-nitrophenol, Appl. Catal., A, 313 (1) (2006), pp. 41-48.

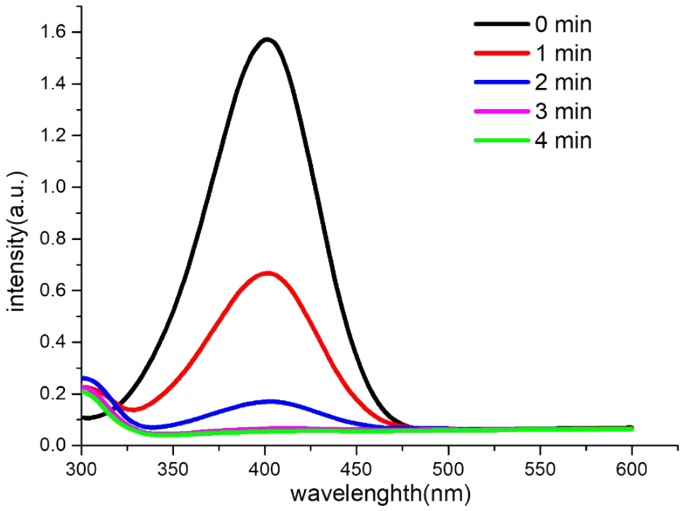

S12 High concentration freely suspended follower-like Au-AgNC nanocrystals (~6 substrates) as catalysts for 4-NP reduction. The extinction spectra of 4-NP showed that the reaction can be completed in about 3 mins.

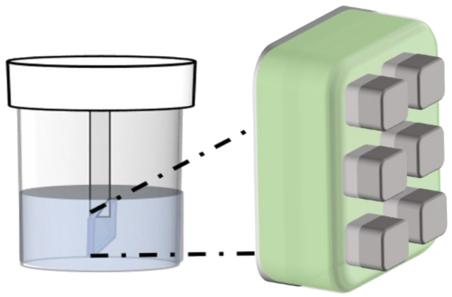

S13 Schematic of the setup used to synthesis bi-component nanocrystals, which partially embedded AgNC as “seeds”.
